# RBM15-dependent m6A modification mediates progression of non-small cell lung cancer cells

**DOI:** 10.1186/s10020-024-01018-z

**Published:** 2024-12-23

**Authors:** Man Wang, Yujiao Qin, Xiaoqi Ai, Xiuhua Liu

**Affiliations:** Department of Respiratory Medicine, The First Affiliated Hospital of Jilin University, 1 Xinmin Street, Changchun, 130021 Jilin China

**Keywords:** RBM15, NSCLC, KLF1, TRIM13, ANXA8

## Abstract

**Background:**

Non-small cell lung cancer (NSCLC) is the predominant form of lung cancer, contributing significantly to global health and economic challenges. This study elucidated the role of RBM15 in NSCLC progression through its involvement in m6A modifications.

**Methods:**

RBM15 levels in NSCLC tissues and cells were assessed via RT-qPCR and Western blotting. The impact of RBM15 knockdown on NSCLC proliferation, invasion, and migration was evaluated using CCK-8, colony formation, and Transwell assays. Expression levels of KLF1, TRIM13, and ANXA8 were determined by RT-qPCR and Western blot. m6A methylation levels were analyzed, while RIP and MeRIP assays were employed to explore the interaction between YTHDF1/YTHDF2/m6A and KLF1/TRIM13, as well as KLF1 binding to the ANXA8 promoter. The ubiquitination of ANXA8 was examined through ubiquitination assays. Xenograft and metastasis models were utilized to assess RBM15’s role in vivo.

**Results:**

RBM15 was found to be overexpressed in NSCLC. Silencing RBM15 led to decreased cell proliferation, invasion, and migration of NSCLC cells. RBM15 upregulated KLF1 and downregulated TRIM13 via YTHDF1/YTHDF2, resulting in the promotion of ANXA8 expression. KLF1 overexpression or TRIM13 downregulation partially reversed the suppressive effects of RBM15 knockdown on NSCLC cell proliferation. ANXA8, upregulated in NSCLC, mitigated the inhibitory effects of RBM15 silencing on malignant behaviors. In vivo*,* RBM15 downregulation hindered NSCLC cell proliferation and metastasis by modulating the KLF1-TRIM13/ANXA8 axis.

**Conclusion:**

RBM15-mediated m6A methylation enhances KLF1 expression and suppresses TRIM13 via YTHDF1/YTHDF2, thereby promoting ANXA8 and facilitating NSCLC progression. These findings provide novel insights and potential therapeutic targets for NSCLC treatment.

## Background

Lung cancer (LC) remains a significant global health challenge, with non-small cell lung cancer (NSCLC) accounting for 80% to 85% of all cases (Chen et al. 2022a). In 2020, lung cancer was responsible for an estimated 1.8 million deaths worldwide, a figure projected to rise, further exacerbating the societal and economic burden (Sung et al. 2021). While smoking is the primary risk factor, others include secondhand smoke, air pollution, genetic predisposition, and environmental exposures (Molina et al. 2008). Early-stage NSCLC is typically managed with complete surgical resection, and treatments such as platinum-based adjuvant chemotherapy, targeted therapy, and immunotherapy have contributed to extending overall survival (OS). However, chemotherapy often comes with severe toxicity, and the development of other alternative therapeutic modalities remains limited (Chaft et al. 2022). This highlights the urgent need for new biomarkers to develop more effective and less toxic treatments for NSCLC.

N6-methyladenosine (m6A) is the most prevalent internal co-transcriptional modification in eukaryotic RNA, regulated by m6A methyltransferases, including methyltransferase-like (METTL) 3/14/16, KIAA1429, ZC3H3, and RNA binding motif (RBM)15. The recognition of m6A sites is facilitated by m6A binding proteins such as the YTH domain family proteins (YTHDF1/2/3) (Jiang et al. 2021). m6A RNA methylation is crucial in regulating both physiological and pathological processes, making it a promising target for cancer therapy (An and Duan 2022). Elevated m6A modification levels are frequently observed in NSCLC, correlating with drug resistance, immune evasion, tumor growth, and metastasis (Jin et al. 2020; Liu et al. 2021; Xie et al. 2022). Methyltransferases catalyze m6A modification, and m6A binding proteins regulate downstream gene expression and RNA metabolism by binding to m6A sites, thereby contributing to cancer progression (He et al. 2019). Among the m6A methyltransferases, RBM15 has been implicated in promoting cancer progression through m6A modification in various cancers, including laryngeal squamous cell carcinoma, pancreatic cancer, and colorectal cancer (Dong et al. 2023; Wang et al. 2021; Zhang et al. 2022). Notably, LC exhibits upregulated RBM15 expression, and RBM15 suppression hinders LC cell proliferation, invasion, and migration (Feng et al. 2023). Furthermore, increased RBM15 protein expression has been observed in lung adenocarcinoma (LUAD) and lung squamous cell carcinoma (LUSC), where it is associated with poor prognosis (Zhang et al. 2020; Zhao et al. 2021). Based on these findings, we hypothesize that RBM15 plays a pivotal role in the malignant progression of NSCLC. Investigating how RBM15 interacts with m6A binding proteins to regulate downstream gene expression is essential for understanding the molecular mechanisms driving NSCLC progression.

Kruppel-like factor 1 (KLF1) is a transcription regulatory factor characterized by a DNA binding domain composed of three similar C_2_H_2_ zinc fingers located at its C-terminus. KLF1 is instrumental in regulating chromatin configuration, transcription initiation, and elongation, thereby activating or repressing target genes (Yien and Bieker 2013). Broadly, KLF1 functions as an oncogenic factor. For instance, KLF1 knockdown has been shown to suppress proliferation, metastasis, and invasion in cervical cancer cells (Zhu et al. 2018). In gastric cancer, KLF1 overexpression promotes cancer cell migration and epithelial-mesenchymal transition (EMT) (Li et al. 2021). Notably, KLF1 is implicated in the M2 polarization of macrophages in NSCLC, facilitating metastasis and the EMT process in these cancer cells (Chen et al. 2022b). Furthermore, KLF1 expression is known to be influenced by the loss of m6A methylation marks (Kuppers et al. 2019). In colorectal cancer, KLF1 expression is upregulated by RBM15 through m6A modification (Chen 2023). This suggests that KLF1 may contribute to NSCLC progression through RBM15-mediated m6A modification, thereby activating downstream genes.

KLF1 also plays a role in the activation of downstream genes (Eaton et al. 2008). Among these, the tripartite-motif (TRIM) family proteins are noteworthy. TRIM proteins, characterized by an N-terminal RING domain, one or two B-box motifs, a coiled-coil domain, and a variable C-terminal domain, are canonical E3 ubiquitin ligases (Huang et al. 2022). These proteins are pivotal in various biological processes, including innate immunity, tumorigenesis, cell differentiation, and development (Zhan and Zhang 2021). TRIM13, a member of the TRIM family, is regulated by demethylases in inflammatory responses via m6A modification (Hu et al. 2024). We hypothesize that in NSCLC, the m6A modification level of TRIM13 may also be regulated by the methyltransferase RBM15.

There is growing evidence to support the tumor suppressor role of TRIM13. For example, TRIM13 expression is significantly reduced in renal cell carcinoma tissues, and its ectopic expression decreases the migration and invasion of renal cell carcinoma cells (Li et al. 2020). In LUAD, TRIM13 inhibits tumor growth by enhancing apoptosis and oxidative stress (Yu et al. 2023). Similarly, TRIM13 expression is downregulated in NSCLC, and its overexpression suppresses tumor growth and induces cell apoptosis (Xu et al. 2019). As a ubiquitin ligase, TRIM13 regulates the protein levels of downstream factors (Li et al. 2022) and functions as a tumor suppressor through ubiquitination (Tomar and Singh 2014). However, the detailed mechanisms of how TRIM13 exerts its tumor-suppressive effects, particularly in the context of m6A modification, remain underexplored.

Annexins are a family of calcium-dependent phospholipid-binding proteins that interact with negatively charged phospholipids in a Ca^2+^-dependent manner. Among these, Annexins A (ANXA) is prominently expressed in vertebrate cells (Xi et al. 2020). The different members of the ANXA protein family play varying roles in tumor proliferation, with some acting as tumor suppressors and others as oncogenes. For instance, downregulation of ANXA1 has been shown to promote the proliferation, invasion, and migration of nasopharyngeal carcinoma cells (Liu et al. 2014). ANXA5, when overexpressed, inhibits the proliferation of cervical cancer cells by regulating the expression of B cell lymphoma-2 (BCL2) and BCL2-associated X (Li et al. 2018).

On the other hand, certain ANXA proteins are identified as oncogenes. ANXA3, for example, is significantly upregulated in hepatocarcinogenesis and promotes both the growth and chemoresistance of hepatocarcinogenesis cells (Guo et al. 2021). Similarly, silencing ANXA2 effectively inhibits the invasion, migration, and tumorigenic potential of hepatocarcinogenesis cells (Zhang et al. 2013). In renal cancer cells, YTHDC1 has been shown to bind to ANXA1 and reduce its mRNA stability through m6A modification (Li et al. 2022) and ANXA2 in renal clear cell carcinoma has been associated with m6A modification levels (Miao et al. 2023). Among the numerous members of the ANXA protein family, we focus on ANXA8, which has been demonstrated to promote tumorigenesis in NSCLC (Lu et al. 2022). However, to date, no studies have explored the involvement of ANXA8 in NSCLC through m6A modification.

In our study, preliminary research has indicated that KLF1 and TRIM13 interact with ANXA8. Based on this, we hypothesize that RBM15 may regulate the expression of KLF1 and TRIM13 through m6A modification, thereby influencing ANXA8 and contributing to the invasion and migration of NSCLC cells both in vivo and in vitro. This perspective could provide novel theoretical insights for the treatment of NSCLC.

## Materials and methods

### Ethics statement

All individual participants in the study provided informed consent, and all procedures involving human participants were conducted in accordance with the ethical standards of The First Affiliated Hospital of Jilin University, following the principles outlined in the Declaration of Helsinki. Animal experiments were performed under the guidance of the Animal Care and Use Committee of The First Affiliated Hospital of Jilin University. The procedures complied with the Guide for the Care and Use of Laboratory Animals by the National Institutes of Health (National Research Council (US) 2011).

### Collection of clinical samples

A total of 50 cases of NSCLC patients who underwent surgical resection at our hospital were enrolled in this study. Both LC tissues and adjacent normal tissues were collected from these patients. The diagnosis of NSCLC was confirmed through histopathological examination. Immediately after resection by professional researchers, all tissues used for analysis were promptly frozen in liquid nitrogen and stored at − 80 ℃ for preservation. The clinicopathological characteristics of the patients are detailed in Table 1. None of the patients received chemotherapy or radiotherapy prior to surgery.

**Table 1:** Relationship between RBM15 expression and clinical variables among NSCLC patients

Characteristic	Number (Total n = 50)	RBM15 expression		*P* value
		Low (n = 25)	High (n = 25)	
Age (years)				
≤ 60	32	15	17	0.556
> 60	18	10	8	
Sex				
Male	35	19	16	0.355
Female	15	6	9	
Lymph node metastasis				
Negative	30	14	16	0.564
Positive	20	11	9	
Histological grade				
Middle to low	25	14	11	0.396
High	25	11	14	
Tumor size (cm)				
≤ 3	28	18	10	0.023
> 3	22	7	15	
TNM stage				
I and II	25	16	9	0.048
III and IV	25	9	16	
Histological classification				
Squamous cell carcinoma	27	15	12	0.395
Adenocarcinoma	23	10	13	
History of smoking				
Never	23	12	11	0.777
Ever	27	13	14	

The comparison between groups was conducted using the Pearson's test; *p* < 0.05 indicates statistically significant differences

### Cell culture

Five human NSCLC cell lines (A549, NCI-H1299, NCI-H1975, NCI-H358, and SPC-A-1) and one human normal bronchial epithelial cell line (16HBE) were obtained from the Shanghai Cell Bank of the Chinese Academy of Sciences. After resuscitation, A549, SPC-A-1, and 16HBE cells were cultured in Dulbecco's Modified Eagle Medium (DMEM; HyClone, Logan, UT, USA), while NCI-H1299, NCI-H1975, and NCI-H358 cells were cultured in Roswell Park Memorial Institute (RPMI)-1640 medium (HyClone). Both media were supplemented with 10% fetal bovine serum (FBS, Invitrogen, Carlsbad, CA, USA). The cells were maintained in a humidified incubator at 37 ℃ with 5% CO_2_ and 95% air.

### Cell treatment

For gene overexpression and silencing studies, plasmids and small interfering RNAs (siRNAs) were designed and synthesized by GenePharma (Shanghai, China). Overexpression constructs included pcDNA3.1 vectors encoding KLF1 (oe-KLF1) and ANXA8 (oe-ANXA8), along with a corresponding control vector (oe-NC). For gene knockdown, siRNAs targeting RBM15 (si-RBM15), TRIM13 (si-TRIM13), YTHDF1 (si-YTHDF1), and YTHDF2 (si-YTHDF2) were used, with a non-targeting siRNA serving as the control (si-NC). NSCLC cells were seeded at 1 × 10^5^ cells per well in 6-well culture plates before transfection. Transfections were performed using Lipofectamine 3000 (Invitrogen) according to the manufacturer's protocol. Cells were analyzed 48 h post-transfection.

### Cell counting kit-8 (CCK-8) assay

NSCLC cells were seeded into 96-well plates at 3 × 10^3^ cells per well. After incubation for 0, 24, 48, or 72 h, 100 μL of CCK-8 solution (Dojindo, Kumamoto, Japan) was added to each well, and the plates were incubated at 37 ℃ for 1 h. The absorbance was then measured at 450 nm using an enzyme-linked immunosorbent assay (ELISA) reader (Thomas Scientific, NJ, USA), allowing for the assessment of cell viability and proliferation.

### Colony formation assay

For the colony formation assay, 500 cells were seeded into each well of 6-well plates and cultured in an appropriate culture medium supplemented with 10% FBS. The medium was refreshed every 4 days. After two weeks, colonies were fixed in 4% paraformaldehyde for 30 min and stained with 0.1% crystal violet (Sigma-Aldrich, St. Louis, MO, USA) in phosphate-buffered saline for 15 min. Colonies containing more than 50 cells were counted under a microscope to determine colony formation efficiency.

### Transwell assay

Cell migration and invasion were assessed using Transwell chambers (Corning, New York, NY, USA) with an 8 μm pore size. For the invasion assay, the upper chamber was coated with Matrigel to simulate the extracellular matrix. A total of 3 × 10^4^ cells were suspended in 100 μL of serum-free medium and added to the apical chamber, while 600 μL of medium containing 10% FBS was added to the basolateral chamber. For the migration assay, 1 × 10^5^ cells were added to the apical chamber without Matrigel coating. After 24 h of incubation, cells that had migrated or invaded through the membrane were fixed with 4% paraformaldehyde for 15 min, stained with 0.1% crystal violet for 20 min, and counted under a microscope. The number of cells on the underside of the membrane represented the extent of cell migration or invasion.

### m6A quantitative analysis

Total RNA was extracted from cells using the TRIzol Reagent (Invitrogen) following standard protocols. To measure the relative m6A content in total RNA, the EpiQuikTM m6A RNA Methylation Quantification Kit (EpiGentek, Farmingdale, NY, USA) was utilized according to the manufacturer's instructions. Briefly, 200 ng of RNA was added to each well of a microplate, followed by the addition of a solution containing m6A-specific antibodies. The absorbance at 450 nm was measured using a microplate reader (BioTek ELx800, BioTek Winooski, Vermont, USA), and the level of m6A was quantified using a colorimetric assay.

### RNA immunoprecipitation (RIP) assay

The RIP assay was conducted using the Magna RIP™ RNA-Binding Protein Immunoprecipitation Kit (Millipore, Billerica, MA, USA) according to the manufacturer's protocol. Cells were first cross-linked with 0.3% formaldehyde and then quenched with a glycine solution (Millipore). Magnetic beads were pre-incubated with antibodies against YTHDF1 (ab220162, Abcam, Cambridge, MA, USA), YTHDF2 (ab220163, Abcam), or control immunoglobulin G (IgG) (ab6757, Abcam) at 25 °C for 30 min. Following antibody binding, cell lysates were incubated with the treated magnetic beads overnight at 4 °C. After extensive washing (six times), RNA–protein complexes were digested with proteinase K buffer at 55 °C for 45 min to release the enriched RNA. The RNA was extracted and analyzed by reverse transcription-quantitative polymerase chain reaction (RT-qPCR) to determine the level of RNA enrichment.

### Methylated RNA immunoprecipitation (MeRIP)

For MeRIP, total RNA was combined with an m6A spike-in control and then added to 300 μL of IP buffer (50 mM Tris–HCl, pH 7.4, 150 mM NaCl, 0.1% NP40, 40 U/μL RNase Inhibitor) containing an m6A antibody (ab208577, Abcam). Dynabeads™ M-280 IgG suspension (Invitrogen) was blocked with freshly prepared 0.5% bovine serum albumin at 4 °C for 2 h and then washed three times with 300 μL of IP buffer. The blocked beads were resuspended in the RNA-antibody mixture, allowing the RNA to bind to the m6A antibody-coated beads. This mixture was incubated at 4 °C for 2 h. After incubation, the beads were washed, and the enriched RNA was eluted with 200 μL of elution buffer at 50 °C for 1 h. The eluted RNA was subsequently subjected to RT-qPCR analysis to determine the levels of m6A-modified RNA.

### RNA stability assay

To assess RNA stability, Actinomycin D (5 μg/mL) (Sigma-Aldrich) was added to the cells to inhibit transcription. Total RNA was isolated at designated time points post-treatment, and glyceraldehyde-phosphate dehydrogenase (GAPDH) was used as an internal control for normalization during the analysis.

### Chromatin immunoprecipitation (ChIP)

ChIP analysis was performed using the MagnaChIP kit (Millipore) following the manufacturer's protocol. NSCLC cells were fixed with 1% formaldehyde to cross-link proteins to DNA, then lysed on ice using ChIP lysis buffer. The lysate was sonicated to shear the chromatin into smaller fragments. The resulting supernatant was incubated with Dynabeads Protein G and a primary antibody against KLF1 (MBS421048, MyBioSource, San Diego, CA, USA). IgG (ab48386, Abcam) was used as a negative control. The enriched DNA–protein complexes were washed three times and analyzed using RT-qPCR.

### Dual-luciferase assay

The ANXA8 promoter region, both wild-type (WT) and mutant (MUT), was amplified and cloned into the pmirGLO dual-luciferase reporter vector (Promega, Madison, WI, USA). The resulting recombinant vectors were named ANXA8-WT and ANXA8-MUT, respectively. These vectors were co-transfected with either oe-KLF1 or oe-NC into cells plated in a 12-well plate. After 48 h of incubation, luciferase activity was measured using the dual luciferase reporter assay system (Promega) according to the manufacturer's instructions. Renilla luciferase activity was used as an internal control to normalize firefly luciferase activity.

### Co-immunoprecipitation (Co-IP)

For Co-IP, cells were lysed in a buffer containing 150 mmol/L NaCl, 50 mmol/L Tris–HCl (pH = 7.6), 1% NP-40, 1 mmol/L EDTA, 1 mmol/L NaF, 1 mmol/L Na_3_VO_4_, 1 mmol/L phenylmethanesulfonyl fluoride (PMSF), and 0.1% protease inhibitor cocktail. Following lysis, the samples were centrifuged, and the supernatant was collected. The supernatant was incubated with a TRIM13 antibody (ab234847, Abcam) or control IgG antibody (ab133470, Abcam) at 4 ℃ for 30 min. Protein A/G plus agarose beads (Santa Cruz Biotechnology, Santa Cruz, MA, CA) were then added and incubated overnight at 4 ℃. After three washes, the precipitated proteins were denatured by boiling in sodium dodecyl sulfate (SDS) buffer loading buffer, followed by Western blot to detect ANXA8 protein levels.

### Ubiquitination assay

Cells were treated with 20 μM MG132 (MCE) for 4 h to inhibit proteasomal degradation, then collected and lysed using the previously described lysis buffer. The lysates were incubated with an ANXA8 antibody (ab111693, Abcam) or a control IgG antibody (ab133470, Abcam). Following this, protein A/G plus agarose beads were added, and the mixture was incubated overnight at 4 ℃. The levels of ANXA8 ubiquitination were subsequently assessed using Western blot analysis with an anti-ubiquitin antibody (ab19247, Abcam).

### Tumor formation in vivo

Male BALB/c nude mice (4 weeks old, 18–20 g) were obtained from the Shanghai Branch of Beijing Vital River Laboratory Animal Technology Co., Ltd. (SYXK (Hu) 2022-0018). The mice were housed under standard conditions and received care in accordance with the experimental protocol. After a one-week acclimation period, the mice were randomly assigned to experimental groups based on body weight, using a random number method recorded by the experimenters. The groups included sh-NC, sh-RBM15, sh-RBM15 + oe-NC, sh-RBM15 + oe-KLF1, sh-RBM15 + sh-NC, and sh-RBM15 + sh-TRIM13.

Lentiviral vectors expressing sh-RBM15, oe-KLF1, or sh-TRIM13, as well as corresponding control vectors (sh-NC, oe-NC), were synthesized and packaged by Genechem. A549 cells were seeded in a 6-well plate and infected with lentivirus at a multiplicity of infection (MOI) of 50. Stable cell lines expressing the transgenes were selected using 2 μg/mL puromycin. For the tumor formation assay, 1 × 10^6^ stably transduced cells were subcutaneously injected into the flanks of the mice in 0.2 mL of phosphate-buffered saline (PBS). Tumor size was measured every 5 days starting from day 10, and the tumor volume was calculated using the formula: width^2^ × length × 0.5. After 30 days, all mice were euthanized with an intraperitoneal injection of 150 mg/kg pentobarbital sodium. Tumors were then collected, weighed, photographed, and processed for further analysis.

For the lung/liver metastasis model, A549 cells (5 × 10^6^) were injected into the tail vein of the nude mice in 0.2 mL of PBS. After 5 weeks, the mice were euthanized, and their lungs and livers were harvested. Tumor nodules formed in the lungs and livers were counted and analyzed. Investigators responsible for the analysis were blinded to the group assignments.

### Immunohistochemistry (IHC) staining

Tumors excised from the mice were fixed in 4% paraformaldehyde overnight. The following day, the tissues were dehydrated, embedded in paraffin, and sectioned into 5 μm thick slices. The sections were deparaffinized using xylene, rehydrated through graded ethanol, and treated with 3% H_2_O_2_ to quench endogenous peroxidase activity. After antigen retrieval and blocking, the sections were incubated overnight with an anti-Ki-67 antibody (ab16667, Abcam) at 4 ℃. The next day, the sections were washed and incubated with a secondary antibody (ab205718, Abcam). Visualization was achieved using 3,3'-diaminobenzidine substrate. The stained sections were photographed under an optical microscope and analyzed by investigators blinded to the experimental groups.

### Hematoxylin and Eosin (H&E) staining

Mouse lung and liver tissues were fixed in 4% paraformaldehyde, followed by dehydration in a series of graded ethanol solutions. The tissues were then embedded in paraffin and sectioned into 5 μm thick slices. The sections were stained with H&E using a kit from Beyotime (Shanghai, China). Following staining, the sections were dehydrated through graded alcohol solutions, cleared in xylene, and sealed with neutral gum. After mounting, the sections were examined and photographed under an optical microscope. The analysis was conducted in a double-blind manner to ensure unbiased results.

### RT-qPCR

Total RNA was extracted using the TRIzol reagent (Invitrogen) according to the manufacturer's protocol. First-strand complementary DNA (cDNA) synthesis was performed using a reverse transcription kit (Thermo Fisher Scientific, Rockford, IL, USA). Real-time PCR was carried out on an ABI 7500 series PCR instrument (Applied Biosystems, Foster City, CA, USA) using SYBR Green Master Mix (Thermo Fisher Scientific). GAPDH was used as a reference gene for normalization of gene expression. Relative expression levels were calculated using the 2^−ΔΔCt^ method (Livak and Schmittgen 2001). The primer sequences used in the study are listed in Table 2.

**Table 2:** Primer sequences for RT-qPCR

Gene	Sequence (5′-3′)
RBM15	F: GCCTTCCCACCTTGTGAGTT
	R: TCAACCAGTTTTGCACGGAC
YTHDF1	F: CGTGGACACCCAGAGAACAA
	R: CGCTCATTGAGGGGTAACTGT
YTHDF2	F: CAGGCATCAGTAGGGCAACA
	R: TTATGACCGAACCCACTGCC
KLF1	F: TCCCCCTCCTTCCTGAGTTG
	R: GGTCTCGGCTATCACACCTG
TRIM13	F: ATCTGTGCTACTCGTGGGGA
	R: AGCTCTGGAAGAGGGACTCA
ANXA8	F: CTCCCACTTCAACCCAGACC
	R: GAAGGACTTGGCGATCTGCT
GAPDH	F: GGTCCCAGCTTAGGTTCATCA
	R: AATCCGTTCACACCGACCTT
ANXA8 promoter	F: CCTGCCAGAGAGTTGTGTAA
	R: AGGGCAGCGCTACACCTGCCT

### Western blot assay

Total protein was extracted using radioimmunoprecipitation assay (RIPA) lysis buffer (Aspen Biotechnology, Wuhan, China). The protein concentration was quantified using the bicinchoninic acid assay kit. Proteins were separated on a 10% SDS–polyacrylamide gel (SDS-PAGE) and transferred onto a polyvinylidene fluoride (PVDF) membrane (Bio-Rad, China). The membrane was blocked and incubated overnight at 4 °C with the appropriate primary antibodies. The next day, the membrane was incubated with a secondary antibody for 2 h at room temperature. After washing with Tris-buffered saline with Tween, protein bands were visualized using an enhanced chemiluminescence kit. The grayscale values of the bands were quantified using NIH Image J software (version 1.52a; National Institutes of Health, Bethesda, Maryland, USA). The following primary antibodies were used in this study: RBM15 (ab96544, 1:500, Abcam), YTHDF1 (ab220162, 1:1000, Abcam), YTHDF2 (ab220163, 1:1000, Abcam), KLF1 (ab313446, 1:1000, Abcam), TRIM13 (ab234847, 1:1000, Abcam), ANXA8 (ab111693, 1:500, Abcam), Tubulin (PA5-58711, 1:500, ThermoFisher). The secondary antibody used was: Anti-rabbit IgG (ab205718, 1:2000, Abcam).

### Bioinformatics analysis

The expression levels of RBM15 in NSCLC were analyzed using the StarBase database (https://rnasysu.com/encori/panGeneDiffExp.php) (Li et al. 2014) and the UALCAN database (https://ualcan.path.uab.edu/analysis.html) (Chandrashekar et al. 2022). The binding sites of KLF1 on the ANXA8 promoters were predicted using the JASPAR database (https://jaspar.genereg.net/) (Castro-Mondragon et al. 2022).

### Statistical analysis

All statistical analyses were conducted using SPSS 21.0 statistical software (IBM SPSS Statistics, Armonk, NY, USA) and GraphPad Prism 8.0 software (GraphPad Software Inc., San Diego, CA, USA). The sample size was calculated using Gpower software (α = 0.05, Effect size = 0.5, Power = 0.95), which determined that a total sample size of 45 would be sufficient.

Initial tests for normality and homogeneity of variance tests were performed to ensure that data were normally distributed and exhibited homogeneity of variance. Comparisons between two groups were performed using the Student's *t*-test. For comparisons involving multiple groups, one-way or two-way analysis of variance (ANOVA) was applied, followed by Tukey's multiple comparisons test. All *p* values were two-tailed, with *p* < 0.05 considered statistically significant and *p* < 0.01 considered highly statistically significant.

## Results

### RBM15 is highly expressed in NSCLC

**Fig. 1:**
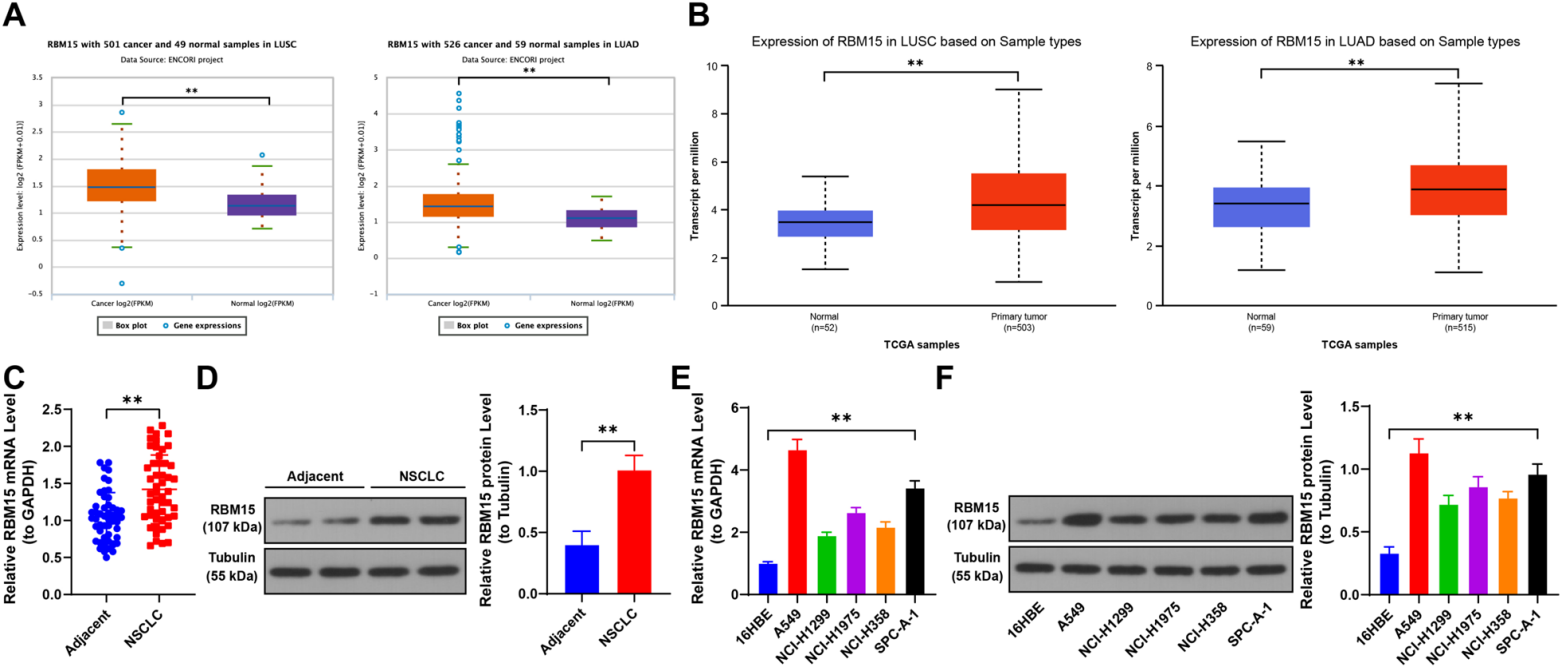
RBM15 is highly expressed in NSCLC. A-B: The expression of RBM15 in lung squamous cell carcinoma and lung adenocarcinoma was predicted using the Starbase and UALCAN databases. C-D: The expression of RBM15 in tumor tissues and adjacent non-tumor tissues (control) in NSCLC patients was detected by RT-qPCR and Western blot assay (representative bands), N = 50; the comparison between the two groups in panels C-D was performed using paired *t*-test. E–F: The expression of RBM15 in different cells was detected by RT-qPCR and Western blot assay, with 16HBE as control, N = 3; the comparisons among multiple groups in panels E–F were conducted using one-way ANOVA, followed by Tukey's multiple comparisons test. The data were presented as mean ± standard deviation. ** *p* < 0.01

Database analysis revealed that RBM15 is significantly upregulated in both LUSC and LUAD (*p* < 0.01, Fig. 1A, B). We further validated this by examining RBM15 expression levels, observing a marked increase in NSCLC cancer tissues compared to normal tissues (*p* < 0.01, Fig. 1C, D). Based on the median expression level of RBM15 in NSCLC cancer tissues, patients were categorized into high and low expression groups. The correlation between RBM15 expression and clinical characteristics was then assessed, showing a significant association with tumor size and tumor-node-metastasis (TNM) stage (Table 1). Additionally, RBM15 expression was significantly elevated in NSCLC cell lines compared to non-cancerous cells (*p* < 0.05, Fig. 1E, F). These findings suggest that RBM15 is upregulated in both NSCLC tissues and cell lines and is associated with key clinical parameters in NSCLC patients.

### Suppression of RBM15 inhibits proliferation, invasion, and migration of NSCLC cells

**Fig. 2:**
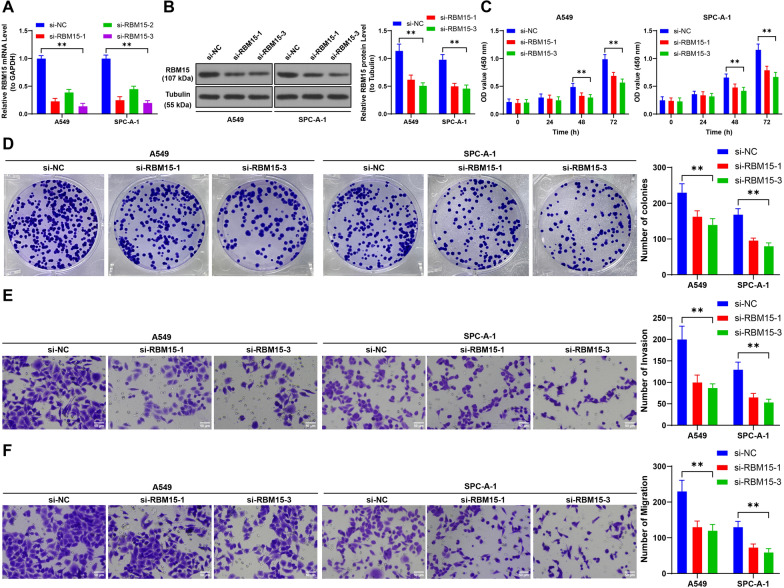
Suppression of RBM15 inhibits proliferation, invasion, and migration of NSCLC cells. Three different siRNAs targeting RBM15 (si-RBM15-1, si-RBM15-2, si-RBM15-3) were separately transfected into A549 and SPC-A-1 cells, with transfection of NC siRNA (si-NC) serving as a negative control. A-B: The expression of RBM15 in the cells was detected by RT-qPCR and Western blot assay. C-D: Cell proliferation was assessed by CCK-8 assay and colony formation assay. E–F: Cell invasion and migration were evaluated using Transwell assay. si-NC was used as control, N = 3. The comparisons among multiple groups in panels A-F were performed using two-way ANOVA, followed by Tukey's multiple comparisons test. The data were presented as mean ± standard deviation. ** *p* < 0.01

To explore the functional role of RBM15 in NSCLC cells, we focused on two cell lines with relatively higher RBM15 expression. We successfully knocked down RBM15 expression using si-RBM15 (*p* < 0.01, Fig. 2A, B), with si-RBM15-1 and si-RBM15-3 demonstrating the most effective interference. The CCK-8 assay results indicated a significant reduction in the proliferation of NSCLC cells following RBM15 knockdown (*p* < 0.01, Fig. 2C). Additionally, RBM15 suppression led to a notable decrease in colony formation, as well as reduced invasion and migration capabilities of the NSCLC cells (*p* < 0.01, Fig. 2D–F). These findings demonstrate that RBM15 downregulation significantly impairs the proliferative, invasive, and migratory potential of NSCLC cells.

### RBM15 mediates m6A modification to upregulate KLF1 and downregulate TRIM13 through YTHDF1 and YTHDF2

**Fig. 3:**
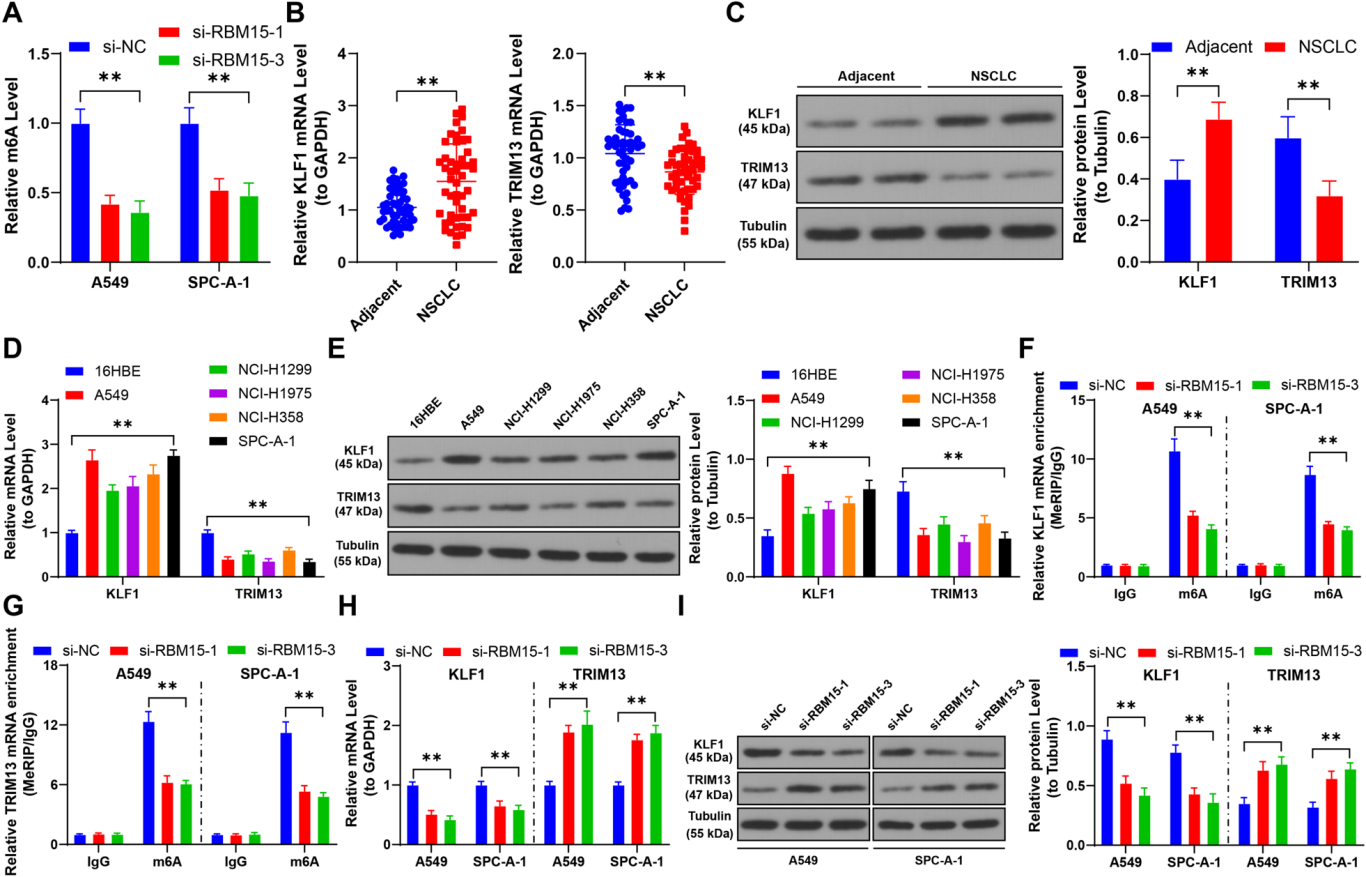
RBM15 mediates m6A modification to upregulate KLF1 and downregulate TRIM13. A: Quantitative analysis of m6A levels in cells, with si-NC as control, N = 3; the comparison among multiple groups in panel A was analyzed using two-way ANOVA, followed by Tukey's multiple comparisons test. B-C: The expression of KLF1 and TRIM13 in tumor tissues and adjacent non-tumor tissues (control) in NSCLC patients was detected by RT-qPCR and Western blot assay (representative bands), N = 50; the comparison between two groups in panels B and C was analyzed using paired* t*-test. D-E: The expression of KLF1 and TRIM13 in different cells was detected by RT-qPCR and Western blot assay, with 16HBE used as control, N = 3; the comparison among multiple groups in panels D and E was analyzed using two-way ANOVA, followed by Tukey's multiple comparisons test. F-G: MeRIP analysis of m6A modification enrichment on KLF1 and TRIM13 mRNA in cells. H-I: The expression of KLF1 and TRIM13 in cells was detected by RT-qPCR and Western blot assay. si-NC was used as control, N = 3; the comparison among multiple groups was analyzed using two-way ANOVA, followed by Tukey's multiple comparisons test. The data were presented as mean ± standard deviation. ** *p* < 0.01

Our investigation revealed that downregulation of RBM15 led to a significant reduction in m6A modification levels in NSCLC cells (*p* < 0.01, Fig. 3A). We also observed that KLF1 expression was elevated, while TRIM13 expression was diminished in NSCLC tissues and cells, findings consistent with previous research (*p* < 0.01, Fig. 3B–E). MeRIP analysis indicated that the mRNA of KLF1 and TRIM13 harbored m6A modifications. However, reducing RBM15 expression resulted in decreased m6A modification levels on KLF1 and TRIM13 mRNA (*p* < 0.01, Fig. 3F, G). Consequently, KLF1 expression decreased, and TRIM13 expression increased following RBM15 downregulation (*p* < 0.01, Fig. 3H, I).

**Fig. 4:**
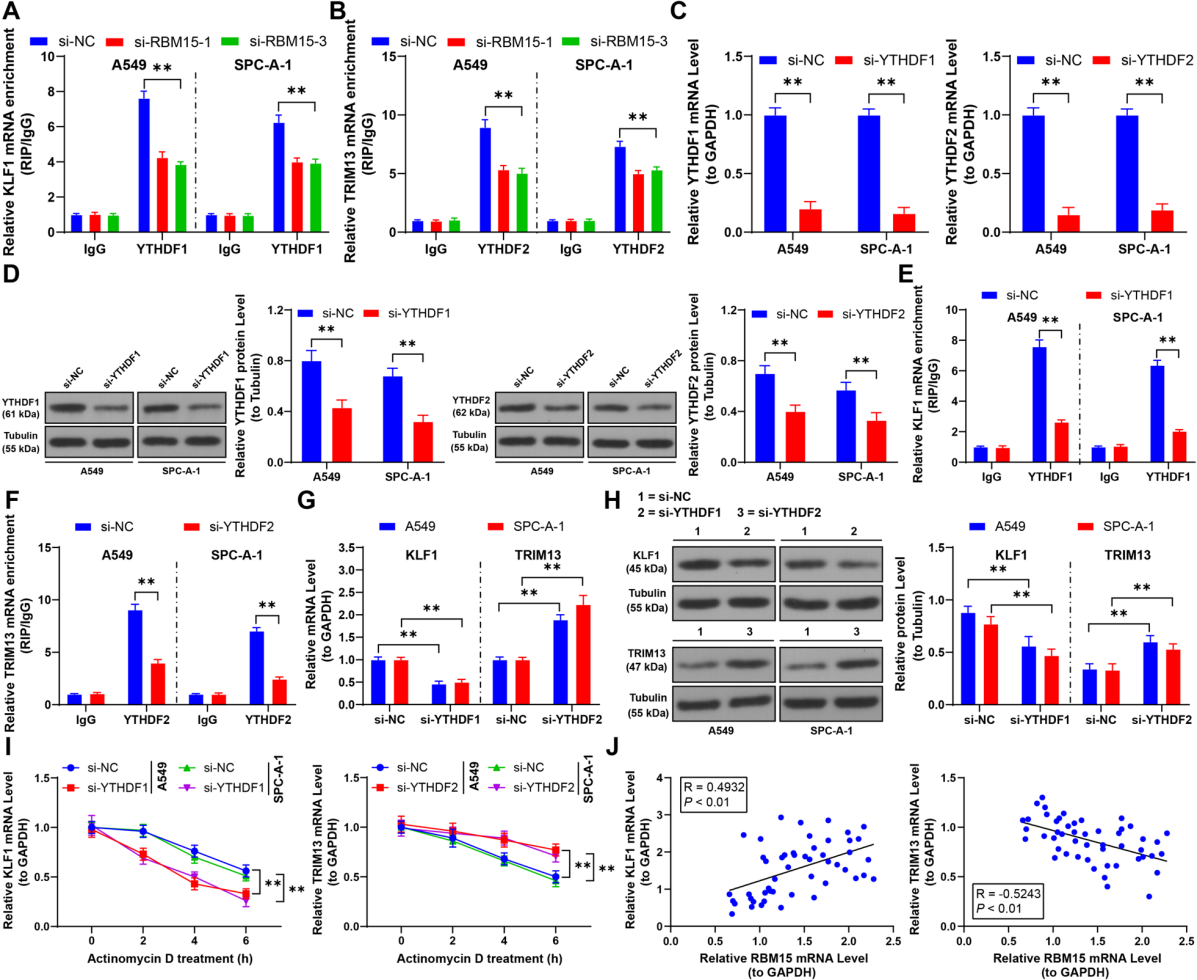
RBM15 mediates m6A modification to upregulate KLF1 or downregulate TRIM13 through YTHDF1 or YTHDF2. A-B: RIP analysis of the enrichment of YTHDF1 or YTHDF2 on KLF1 or TRIM13 mRNA in cells, with si-NC used as control, N = 3; the comparison among multiple groups in panels A and B was analyzed using two-way ANOVA, followed by Tukey's multiple comparisons test; si-YTHDF1 or si-YTHDF2 was separately transfected into A549 and SPC-A-1 cells, with si-NC transfection serving as a negative control. C-D: The expression of YTHDF1 or YTHDF2 was detected by RT-qPCR and Western blot assay, E–F: RIP analysis of the enrichment of YTHDF1 or YTHDF2 on KLF1 or TRIM13 mRNA in cells, G-H: The expression of KLF1 or TRIM13 was detected by RT-qPCR and Western blot assay, I: After treatment with actinomycin D, the stability of KLF1 or TRIM13 mRNA in cells was assessed by RT-qPCR. si-NC was used as control, N = 3; the comparison among multiple groups in panels C-I was analyzed using two-way ANOVA, followed by Tukey's multiple comparisons test. J: The correlation between KLF1 or TRIM13 and RBM15 in NSCLC tumor tissues was analyzed by Pearson correlation analysis, N = 50. The data were presented as mean ± standard deviation. ** *p* < 0.01

Further, RIP analysis showed reduced enrichment of YTHDF1 and YTHDF2 on KLF1 and TRIM13 after RBM15 downregulation (*p* < 0.01, Fig. 4A, B). Subsequent transfection with siRNA to knock down YTHDF1 and YTHDF2 expression confirmed a reduction in their binding to KLF1 and TRIM13 mRNA (*p* < 0.01, Fig. 4C–F). Specifically, YTHDF1 knockdown led to decreased KLF1 expression, while YTHDF2 knockdown resulted in increased TRIM13 expression (*p* < 0.01, Fig. 4G, H). Moreover, YTHDF1 downregulation reduced KLF1 mRNA stability, and YTHDF2 downregulation enhanced TRIM13 mRNA stability (*p* < 0.01, Fig. 4I). In NSCLC cancer tissues, RBM15 expression was positively correlated with KLF1 and negatively correlated with TRIM13 (*p* < 0.01, Fig. 4J). Collectively, these findings suggest that RBM15 mediates m6A modifications to regulate KLF1 and TRIM13 expression through YTHDF1 and YTHDF2.

### Upregulation of KLF1 and downregulation of TRIM13 alleviates the inhibitory effect of RBM15 downregulation on NSCLC cells

**Fig. 5:**
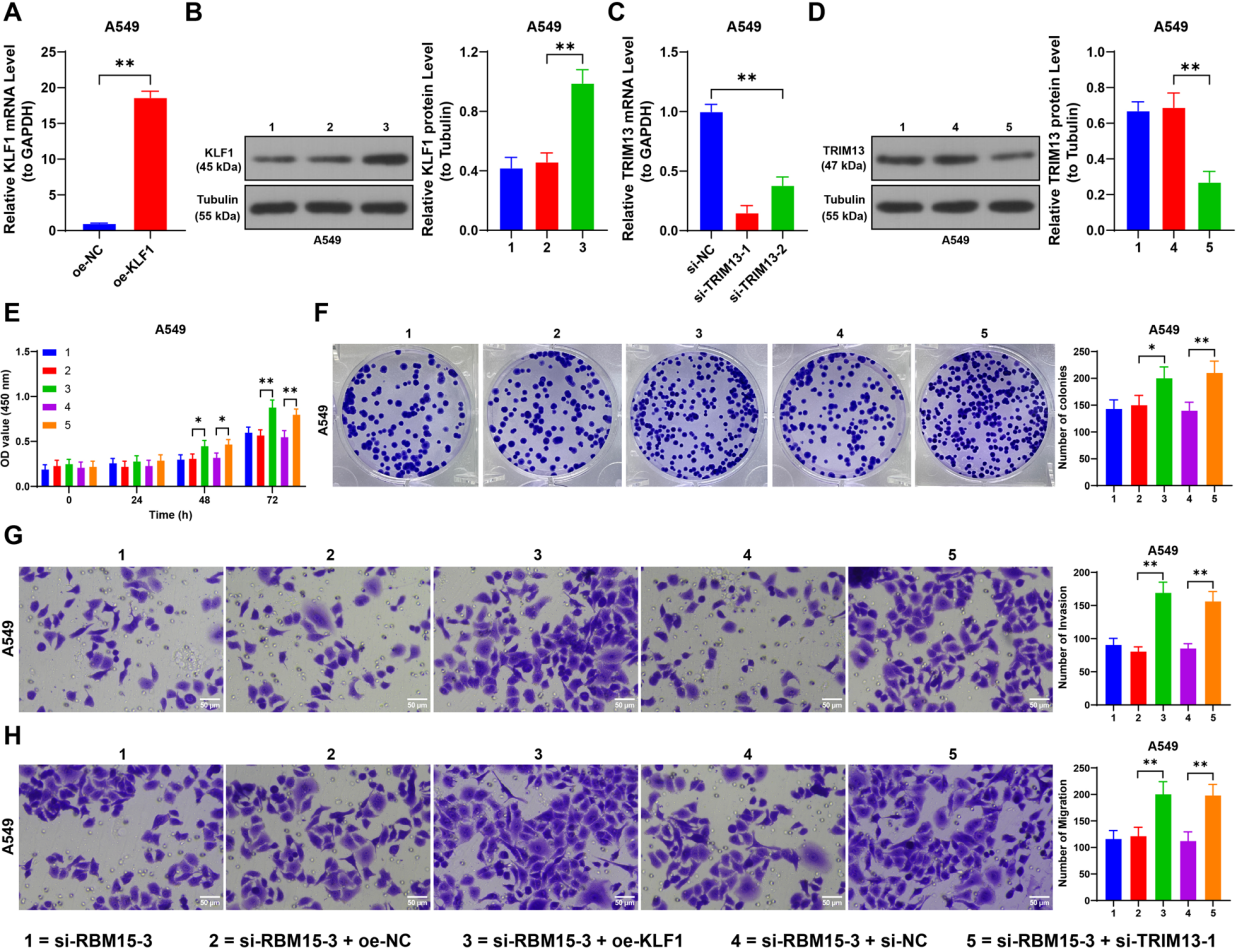
Upregulation of KLF1 or downregulation of TRIM13 alleviates the inhibitory effect of RBM15 downregulation on NSCLC cells. A549 cells were transfected with pcDNA3.1-KLF1 (oe-KLF1), with oe-NC transfection as the negative control. Two strands of si-TRIM13 were transfected into A549 cells, with si-NC transfection as the negative control. A: RT-qPCR was performed to detect the expression of KLF1, with oe-NC as control, N = 3; the comparison between two groups in panel A was analyzed using *t*-test. B-D: RT-qPCR and Western blot analyses were performed to assess the expression levels of KLF1 or TRIM13, with si-RBM15-1 + oe-NC or si-RBM15-1 + si-NC serving as the control groups, N = 3; the comparison of data among multiple groups in panels B-D was analyzed using one-way ANOVA, followed by Tukey's multiple comparisons test. E: Cell proliferation was assessed by CCK-8 assay, with si-RBM15-1 + oe-NC or si-RBM15-1 + si-NC as the control groups, N = 3; the comparison among multiple groups in panel C was analyzed using two-way ANOVA, followed by Tukey's multiple comparisons test. F: Cell proliferation was assessed by colony formation assay, G-H: Cell invasion and migration were evaluated by Transwell assay, with si-RBM15-1 + oe-NC or si-RBM15-1 + si-NC as the control groups, N = 3; the comparison among multiple groups in panels F–H was analyzed using one-way ANOVA, followed by Tukey's multiple comparisons test. The data were presented as mean ± standard deviation. * *p* < 0.05, ** *p* < 0.01

To further understand the role of KLF1 and TRIM13 in NSCLC, we overexpressed KLF1 in A549 cells by transfecting them with oe-KLF1 (*p* < 0.01, Fig. 5A, B) and then combined this with RBM15 knockdown using si-RBM15-3. The results showed that overexpressing KLF1 significantly enhanced cell proliferation and increased colony formation (*p* < 0.01, Fig. 5E, F). Additionally, compared to cells with RBM15 knockdown alone, those in the combined treatment group exhibited heightened invasion and migration capabilities (*p* < 0.01, Fig. 5G, H).

Similarly, we knocked down TRIM13 expression by transfecting A549 cells with si-TRIM13 (*p* < 0.01, Fig. 5C, D) and selected si-TRIM13-1 for further analysis due to its higher knockdown. When combined with RBM15 knockdown, TRIM13 downregulation also promoted cell proliferation, increased colony formation (*p* < 0.01, Fig. 5E, F), and counteracted the reduction in invasion and migration caused by RBM15 downregulation (*p* < 0.01, Fig. 5G, H). These findings indicate that increasing KLF1 expression or decreasing TRIM13 expression can mitigate the inhibitory effects of RBM15 downregulation on NSCLC cells.

### KLF1 and TRIM13 regulate the transcription and protein levels of ANXA8

**Fig. 6:**
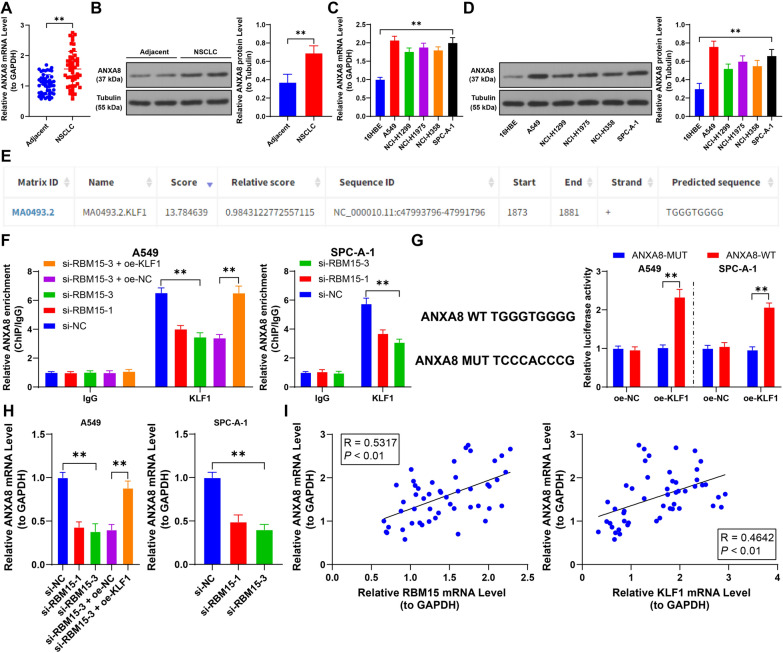
KLF1 promotes the transcription level of ANXA8. A-B: The expression of ANXA8 in tumor tissues and adjacent non-tumor tissues (control) in NSCLC patients was detected by RT-qPCR and Western blot assay (representative bands), N = 50; the comparison between two groups in panels A and B was analyzed using paired *t*-test. C-D: The expression of ANXA8 in different cells was detected by RT-qPCR and Western blot assay, with 16HBE used as control, N = 3; the comparison among multiple groups in panels C and D was analyzed using one-way ANOVA, followed by Tukey's multiple comparisons test. E: The binding sequence of KLF1 to the ANXA8 promoter was predicted by the JASPAR database. F: The enrichment of KLF1 on the ANXA8 promoter in cells was analyzed by ChIP, with si-NC or si-RBM15-3 + oe-NC used as control, N = 3; the comparison among multiple groups in panel F was analyzed using two-way ANOVA, followed by Tukey's multiple comparisons test. G: The binding of KLF1 to the ANXA8 promoter was analyzed by the dual-luciferase reporter assay, with oe-NC used as control, N = 3; the comparison among multiple groups in panel G was analyzed using two-way ANOVA, followed by Tukey's multiple comparisons test. H: The transcription levels of ANXA8 in different cells were detected by RT-qPCR, with si-NC or si-RBM15-3 + oe-NC used as control, N = 3; the comparison among multiple groups in panel H was analyzed using one-way ANOVA, followed by Tukey's multiple comparisons test. I: The correlation between ANXA8 and KLF1 and RBM15 in NSCLC tumor tissues was analyzed by Pearson correlation analysis, N = 50. The data were presented as mean ± standard deviation. ** *p* < 0.01

Our study revealed that ANXA8 was significantly upregulated in NSCLC tissues and cells (*p* < 0.01, Fig. 6A–D). Given that KLF1 has binding sites on the ANXA8 promoter (Fig. 6E), we hypothesized that KLF1 and TRIM13 collectively regulated ANXA8 expression. ChIP analysis confirmed that KLF1 binds to the ANXA8 promoter, and KLF1 overexpression significantly increased its enrichment on the ANXA8 promoter (*p* < 0.01, Fig. 6F). This binding relationship was further validated by a dual-luciferase reporter assay (*p* < 0.01, Fig. 6G). Additionally, RT-qPCR results demonstrated that ANXA8 mRNA levels decreased following RBM15 downregulation, whereas KLF1 overexpression led to increased ANXA8 mRNA levels (*p* < 0.01, Fig. 6H). Moreover, in NSCLC tissues, a positive correlation was observed between ANXA8 expression and both RBM15 and KLF1 (*p* < 0.01, Fig. 6I).

**Fig. 7:**
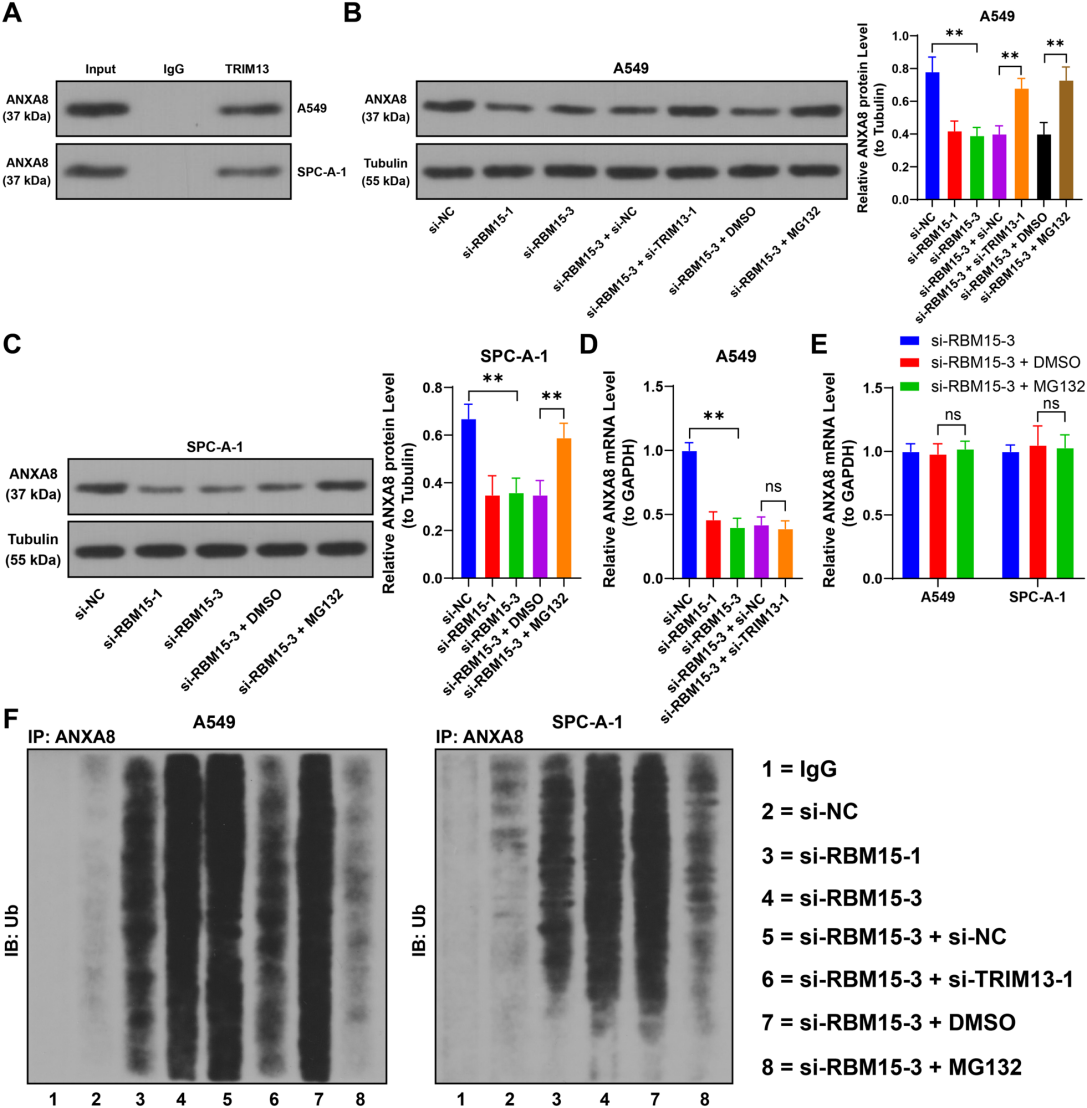
TRIM13 inhibits the protein level of ANXA8 through ubiquitination. A: The interaction between ANXA8 and TRIM13 was analyzed by Co-IP, with IgG used as control, N = 3. B-C: The expression of ANXA8 in different cells was detected by Western blot assay, D: The transcription levels of ANXA8 in different cells were measured by RT-qPCR, with si-NC or si-RBM15-3 + si-NC or si-RBM15-3 + DMSO used as control, N = 3; the comparison among multiple groups in panels B-D was analyzed using one-way ANOVA, followed by Tukey's multiple comparisons test. E: The transcription levels of ANXA8 in different cells were measured by RT-qPCR, with si-RBM15-3 + DMSO used as control, N = 3; the comparison among multiple groups in panel E was analyzed using two-way ANOVA, followed by Tukey's multiple comparisons test. F: The ubiquitination level of ANXA8 was examined by ubiquitination assay, with IgG used as control, N = 3. The data were presented as mean ± standard deviation. ns indicates *p* > 0.05, ** *p* < 0.01

Furthermore, Co-IP analysis confirmed a protein interaction between TRIM13 and ANXA8 (Fig. 7A). Following RBM15 downregulation, ANXA8 protein levels were reduced, whereas TRIM13 downregulation led to an increase in ANXA8 protein levels (*p* < 0.05, Fig. 7B, C). Notably, TRIM13 downregulation did not influence ANXA8 mRNA levels (*p* > 0.05, Fig. 7D). Analysis of ANXA8 ubiquitination revealed that RBM15 downregulation elevated ANXA8 ubiquitination levels, while TRIM13 downregulation diminished them (Fig. 7F). Additionally, treatment with the proteasome inhibitor MG132 enhanced ANXA8 protein expression and lowered its ubiquitination levels (*p* < 0.05, Fig. 7B, C, F), without altering ANXA8 mRNA levels (*p* > 0.05, Fig. 7E). In summary, KLF1 binds to the ANXA8 promoter to activate its transcription, while TRIM13 interacts with the ANXA8 protein, promoting its degradation through ubiquitination.

### Upregulation of ANXA8 alleviates the inhibitory effect of RBM15 downregulation on NSCLC cells

**Fig. 8:**
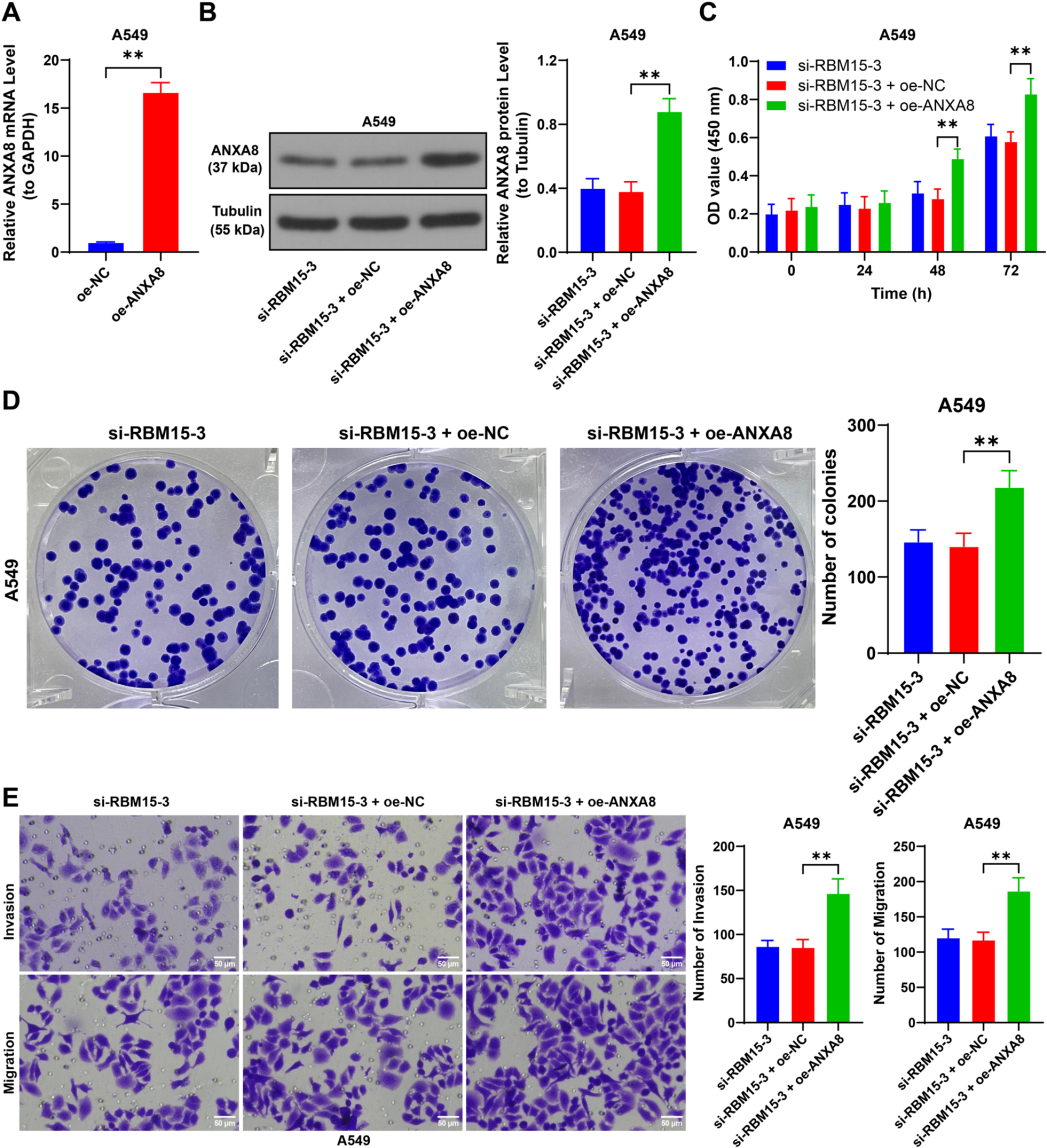
Upregulation of ANXA8 alleviates the inhibitory effect of RBM15 downregulation on NSCLC cells. A549 cells were transfected with oe-ANXA8, with oe-NC transfection as the negative control. A: The expression of ANXA8 was detected by RT-qPCR, with oe-NC used as control, N = 3; the comparison between two groups in panel A was analyzed using *t*-test. B: The expression of ANXA8 was detected by Western blot assay, with si-RBM15-3 + oe-NC used as control, N = 3; the comparison among multiple groups in panel B was analyzed using one-way ANOVA, followed by Tukey's multiple comparisons test. C: Cell proliferation was assessed by CCK-8 assay, with si-RBM15-3 + oe-NC used as control, N = 3; the comparison among multiple groups in panel C was analyzed using two-way ANOVA, followed by Tukey's multiple comparisons test. D: Cell proliferation was detected by colony formation assay, E: Cell invasion and migration were evaluated by Transwell assay, with si-RBM15-3 + oe-NC used as control, N = 3; the comparison among multiple groups in panels D and E was analyzed using one-way ANOVA, followed by Tukey's multiple comparisons test. The data were presented as mean ± standard deviation. ** *p* < 0.01

We transfected A549 cells with oe-ANXA8 to increase ANXA8 expression (*p* < 0.01, Fig. 8A, B) and combined this with si-RBM15-3 treatment to assess ANXA8's role. Compared to the si-RBM15-3 + oe-NC group, the si-RBM15-3 + oe-ANXA8 group exhibited enhanced cell proliferation and a greater number of colonies (*p* < 0.01, Fig. 8C, D). Additionally, ANXA8 overexpression led to increased cell invasion and migration capabilities (*p* < 0.01, Fig. 8E, F). These findings suggest that ANXA8 upregulation mitigates the inhibitory effects of RBM15 downregulation on NSCLC cells.

### Downregulation of RBM15 inhibits tumor growth and lung/liver metastasis in NSCLC cells

**Fig. 9:**
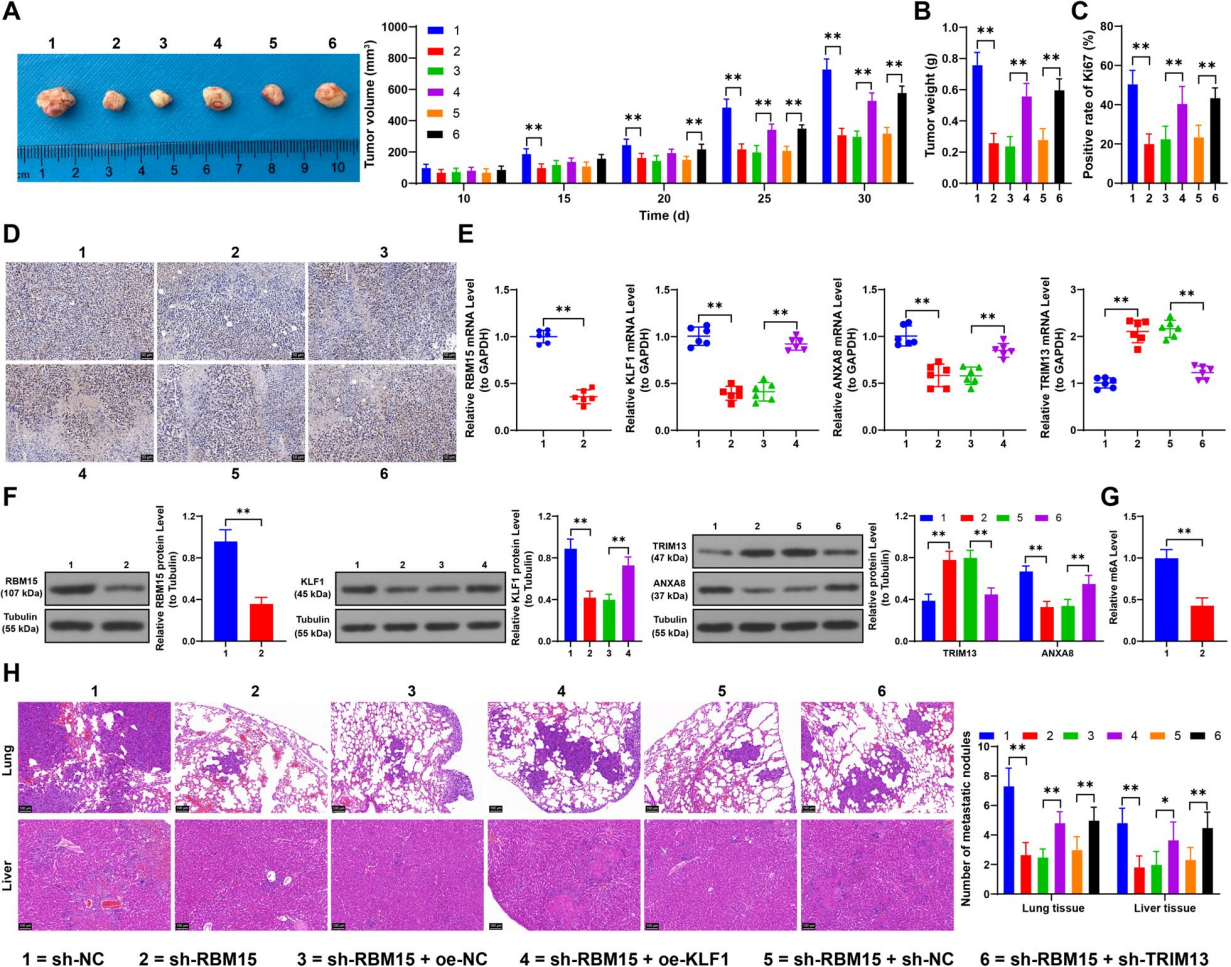
Downregulation of RBM15 inhibits tumor growth and lung/liver metastasis in NSCLC cells. A: After subcutaneous injection of A549 cells in nude mice, tumor size was measured every 5 days; on day 30, the mice were euthanized and tumor tissues, and representative images of the tumor tissues were photographed, with sh-NC, sh-RBM15 + oe-NC, or sh-RBM15 + sh-NC used as control, N=6; the comparison among multiple groups in panel A was analyzed using two-way ANOVA, followed by Tukey's multiple comparisons test. B: Tumor weight was measured, C-D: The positive rate of Ki67 in tumor tissues was detected by immunohistochemical staining, with sh-NC, si-RBM15 + oe-NC, or sh-RBM15 + sh-NC used as control, N = 6; the comparison among multiple groups in panels B and C was analyzed using one-way ANOVA, followed by Tukey's multiple comparisons test. E–F: Expression of RBM15, KLF1, ANXA8, and TRIM13 in tumor tissues was detected by RT-qPCR and Western blot assay, G: Quantification of m6A levels in tumor tissues, with sh-NC, si-RBM15 + oe-NC, or sh-RBM15 + sh-NC used as control, N = 6; the comparisons between two groups in panels E (left), F (left), and G were analyzed using *t*-test; the comparison among multiple groups in panels E and F (center) was analyzed using one-way ANOVA, and in panel F (right) was analyzed using two-way ANOVA, followed by Tukey's multiple comparisons test. H: After the construction of a xenograft metastasis model of A549 cells in nude mice, H&E staining was performed on lung and liver tissues to observe metastasis, with sh-NC, si-RBM15 + oe-NC, or sh-RBM15 + sh-NC used as control, N = 6; the comparison among multiple groups in panel H was analyzed using two-way ANOVA, followed by Tukey's multiple comparisons test. The data were presented as mean ± standard deviation. * *p* < 0.05, ** *p* < 0.01

Finally, we established two in vivo tumor formation models. Our findings revealed that RBM15 downregulation inhibited tumor growth, as evidenced by reduced tumor volume, lower tumor weight, and decreased Ki67 positivity. In contrast, KLF1 overexpression and TRIM13 downregulation accelerated tumor growth (*p* < 0.05, Fig. 9A–D). Analysis of tumor tissues showed significant downregulation of RBM15, KLF1, and ANXA8, along with reduced m6A content (*p* < 0.01, Fig. 9E–G), while TRIM13 expression was increased (*p* < 0.01, Fig. 9E, F) in the sh-RBM15 group compared to the sh-NC group. Compared to the sh-RBM15 + oe-NC group, the sh-RBM15 + oe-KLF1 group exhibited significant increases in KLF1 expression and ANXA8 mRNA levels (*p* < 0.05, Fig. 9E, F). Additionally, compared to the sh-RBM15 + sh-NC group, the sh-RBM15 + sh-TRIM13 group showed decreased TRIM13 expression and ANXA8 protein levels (*p* < 0.05, Fig. 9E, F). Furthermore, our experiments demonstrated that RBM15 downregulation significantly inhibited lung and liver metastasis of tumor cells, while KLF1 overexpression and TRIM13 downregulation significantly promoted lung and liver metastasis (*p* < 0.05, Fig. 9H). In summary, RBM15 regulates the KLF1/TRIM13 axis via YTHDF1/YTHDF2-dependent m6A modification, ultimately promoting in vivo proliferation and metastasis of NSCLC cells.

## Discussion

**Fig. 10:**
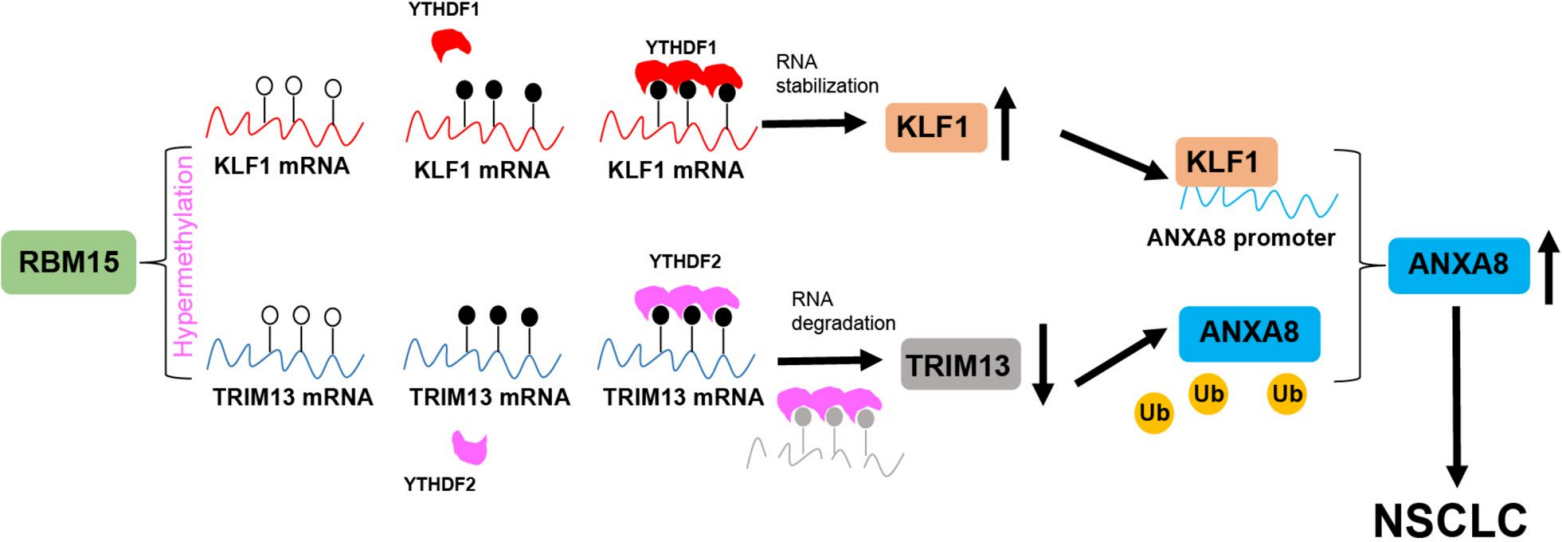
RBM15-dependent m6A modification mediates the progression of non-small cell lung cancer cells. RBM15 mediates m6A methylation to upregulate KLF1 expression through YTHDF1 and downregulate TRIM13 expression through YTHDF2. The highly expressed KLF1 promotes ANXA8 transcription by binding to the ANXA8 promoter, while the downregulation of TRIM13 promotes ANXA8 protein levels through ubiquitination modification, thereby promoting proliferation, invasion, and migration of NSCLC cells

LC remains a leading cause of cancer-related deaths in China, with NSCLC being the most prevalent subtype (Xia et al. 2022). Despite significant advancements in early diagnosis and therapeutic interventions that have markedly improved survival rates and quality of life for many patients (Duma et al. 2019), treatment options for those with advanced, unresectable NSCLC remain limited. Conventional treatments, such as chemotherapy and radiotherapy, continue to pose significant challenges due to their associated toxicities and adverse effects (Alexander et al. 2020). In this study, we explored the role of RBM15 overexpression in NSCLC and found that the RBM15-KLF1/TRIM13-ANXA8 axis plays a crucial role in promoting cell proliferation, invasion, and migration in NSCLC (Fig. 10). These findings suggest that this axis could serve as a novel therapeutic target for NSCLC.

m6A modification has been established as a critical regulator of cellular functions in cancer progression, influencing processes such as RNA splicing, translation, degradation, and decay (Wang et al. 2020). RBM15, in particular, has been shown to play an oncogenic role in various cancers. For instance, in laryngeal squamous cell carcinoma, RBM15 enhances the malignant progression by promoting m6A modification of TMBIM6, thereby increasing TMBIM6 mRNA levels (Wang et al. 2021). Similarly, in cervical cancer, RBM15 inhibition reduces m6A methylation of OTUB2, leading to the inactivation of the AKT/mTOR pathway, which in turn decreases cell proliferation and metastasis capacity (Song and Wu 2023). However, the role of RBM15 in NSCLC has not been extensively studied. Our research demonstrated that RBM15 downregulation inhibits NSCLC tumor growth, reduces Ki67 positivity, and suppresses lung and liver metastasis. These findings indicate that silencing RBM15 may be a key factor in suppressing cancer cell and tumor growth.

Further experiments revealed that RBM15 silencing reduced the enrichment of YTHDF1 and YTHDF2 on KLF1 and TRIM13, leading to decreased expression and mRNA stability of KLF1, and increased expression and mRNA stability of TRIM13. This resulted in the inhibition of NSCLC cell growth. These findings suggest that RBM15 downregulation decreases m6A modification levels, thereby inhibiting NSCLC progression. The role of RBM15 in mRNA stability may be linked to its ability to form liquid-like condensates that partially co-localize with m6A-modified punctate transcripts in the nucleus, facilitating the deposition of m6A on relevant RNA transcripts (Jiang et al. 2022). Additionally, RBM15 knockout disrupts the migration and invasion capabilities of LUAD cells, with RBM15 expression being associated with lymph node metastasis, TNM staging, and histopathology in LUAD (Ma et al. 2022). Our results align with these findings, as we observed that RBM15 is highly expressed in NSCLC and positively correlates with tumor size (> 3 cm) and advanced TNM stage (III and IV). This underscores the potential clinical relevance of RBM15 in NSCLC treatment. Furthermore, recent studies suggest that silencing RBM15 can decrease the expression of TGF-β/Smad2 and increase Fe^2+^ levels and lipid peroxidation in LC cells, promoting ferroptosis and inhibiting cell growth (Feng et al. 2023). This presents a new avenue for future research on the downstream mechanisms of RBM15 in NSCLC.

RBM15 has been shown to enhance the stability and expression of KLF1 mRNA in colorectal cancer through IGF2BP3-mediated m6A modification (Chen 2023). Another study demonstrated that RBM15 regulates the m6A level and stability of TMBIM6 via IGF2BP3, with IGF2BP3 binding to the m6A site in the 3'UTR region of TMBIM6 (Wang et al. 2021). In our study, we observed that YTHDF1, another m6A reader, was downregulated following RBM15 silencing, which subsequently reduced the mRNA stability of KLF1. This study is the first to confirm that RBM15 mediates m6A modification to upregulate KLF1 expression through YTHDF1. YTHDF1 recognizes m6A-modified mRNA and promotes its translation, thereby enhancing protein expression (Chen et al. 2021). Moreover, the Wnt/β-catenin pathway is suppressed by KLF1 silencing in gastric cancer, inhibiting migration and epithelial-mesenchymal transition of gastric cancer cells (Li et al. 2021). Knockdown of KLF1 expression has also been suggested to potentially inhibit the invasion and migration of cervical cancer cells by reducing the expression of Ki67 (Zhu et al. 2018). Notably, positive expression of Ki67 is correlated with the histological type of NSCLC, lymph node metastasis, and TNM staging (Lei et al. 2013). Additionally, KLF1 has been identified as a downstream target of miR-326, with miR-326 mimics suppressing KLF1, thereby inhibiting NSCLC cell migration and inducing apoptosis (Chen et al. 2022b). Given these findings, silencing KLF1 is widely believed to have potential anti-cancer effects. However, in our study, we found that upregulating KLF1 led to increased tumor growth, enhanced proliferation capacity, and promoted lung metastasis and liver metastasis in NSCLC cancer cells. This upregulation also alleviated the inhibitory effects of RBM15 downregulation on NSCLC cancer cell growth.

Our RIP assay revealed that downregulation of RBM15 led to increased expression of TRIM13 and reduced YTHDF2 enrichment on TRIM13. The m6A methylation process facilitated by RBM15 involves recognition by YTHDF2 (Xu et al. 2024). YTHDF2, an m6A reader, targets gene mRNA degradation and may directly bind to the 3'UTR of TRIM7 mRNA to reduce its mRNA stability (Zhou et al. 2020). Our study demonstrated that RBM15 downregulation decreased YTHDF2 expression, thereby alleviating YTHDF2's degradation function and increasing TRIM13 mRNA stability.

TRIM13 expression in breast cancer has been positively correlated with metastasis-free survival rates, with higher levels of TRIM13 associated with better prognosis (Chen et al. 2019). In LUAD, TRIM13 is downregulated and acts by upregulating TRIM13-mediated p62 ubiquitination and degradation, which inhibits LUAD cell proliferation, increases apoptosis, and induces oxidative stress (Yu et al. 2023). Our findings suggested that TRIM13 was also involved in the ubiquitination and degradation of downstream proteins. TRIM13 negatively regulates the NF-κB signaling pathway, and its dysregulation may contribute to cancer pathogenesis (Tomar and Singh 2014). Furthermore, overexpression of TRIM13 in NSCLC has been shown to inactivate the NF-κB pathway, increase cleaved caspase-3 levels, inhibit tumor growth, and induce apoptosis in NSCLC cells (Xu et al. 2019). The specific mechanisms by which TRIM13 interacts with the NF-κB pathway in NSCLC represent a promising direction for future research. Currently, our study demonstrates that TRIM13 downregulation promotes NSCLC cancer cell proliferation, increases clone numbers, enhances lung and liver metastasis, and boosts cancer cell invasion and migration.

ChIP and RT-qPCR analyses have shown that KLF1 overexpression enhances its binding to the ANXA8 promoter, leading to increased ANXA8 mRNA levels. Concurrently, TRIM13 downregulation results in reduced ubiquitination of ANXA8, thereby promoting ANXA8 protein expression. Elevated ANXA8 expression has been implicated in the progression and metastasis of various cancers. For instance, overexpression of ANXA8 has been shown to promote cancer cell migration, invasion, and EMT in bladder cancer (Yuan et al. 2021). In gastric cancer, high ANXA8 expression is closely associated with advanced TNM stage and poor differentiation, indicating a worse prognosis for patients (Ma et al. 2020). Similarly, elevated ANXA8 levels have been observed in ovarian cancer tissues, where it may play a role in cell migration, lymphocyte infiltration, and immune modulation (Gou et al. 2019). Additionally, in NSCLC, long non-coding MELTF Antisense RNA 1 has been shown to directly bind and displace the RNA-binding protein YBX1, which is involved in tumor development, thereby activating ANXA8 transcription and promoting tumorigenesis (Lu et al. 2022). In our study, upregulation of ANXA8 resulted in enhanced proliferation capacity and colony formation of NSCLC cancer cells, as well as increased invasive and migratory capabilities.

However, our study has several limitations. First, we validated the mechanism only at the cellular and animal levels; these preliminary findings are not yet sufficient to support the clinical application of targeting RBM15. Second, our exploration of the downstream mechanisms of RBM15 was relatively narrow, focusing primarily on KLF1 and TRIM13. This limited scope restricts our understanding of the broader impact of RBM15 on various stages of NSCLC development. Third, it remains unclear whether KLF1 and TRIM13 can jointly regulate other factors, which could provide additional insights into the molecular pathways involved in NSCLC progression. Lastly, the potential effects of this mechanism on autophagy, ferroptosis, pyroptosis, and other cellular processes have not been investigated.

Future research should focus on exploring the upstream regulators of RBM15 in NSCLC cells, as well as investigating other downstream targets and mechanisms influenced by RBM15. A comprehensive understanding of RBM15's role in NSCLC could provide a theoretical basis for new therapeutic approaches and offer novel directions for the treatment of this disease.

## Conclusions

In conclusion, RBM15 mediates m6A methylation to upregulate KLF1 expression through YTHDF1 and downregulate TRIM13 expression through YTHDF2. This regulation subsequently enhances the transcription and protein levels of ANXA8, ultimately leading to increased proliferation, invasion, and migration of NSCLC cells.

## Data Availability

The datasets used and analyzed during the current study are available from the corresponding author upon reasonable request.

## Abbreviations

LC: Lung cancer
NSCLC: Non-small cell lung cancer
RBM15: RNA-binding motif protein 15
KLF1: Kruppel-like factor 1
TRIM13: Tripartite motif 13
ANXA8: Annexin A8
m6A: N6-methyladenosine
YTHDF: YTH domain family
LUSC: Lung squamous cell carcinoma
LUAD: Lung adenocarcinoma
OS: Overall survival
METTL3: Methyltransferase-like 3
NLRP1: NLR family pyrin domain-containing 1
FBS: Fetal bovine serum
Si: Small interfering
CCK-8: Cell counting kit-8
ELISA: Enzyme-linked immunosorbent assay
RIP: RNA Immunoprecipitation
IgG: Immunoglobulin G
RT-qPCR: Reverse transcription quantitative polymerase chain reaction
GAPDH: Glyceraldehyde-phosphate dehydrogenase
ChIP: Chromatin immunoprecipitation
WT: Wild-type
MUT: Mutant
Co-IP: Co-Immunoprecipitation
PMSF: Phenylmethanesulfonyl fluoride
SDS-PAGE: Sodium dodecyl sulfate polyacrylamide gel electrophoresis
MOI: Multiplicity of infection
IHC: Immunohistochemistry
H&E: Hematoxylin and eosin
cDNA: Complementary DNA
RIPA: Radioimmunoprecipitation assay
PVDF: Polyvinylidene fluoride
ANOVA: Analysis of variance
